# The Role of ARID5B in Acute Lymphoblastic Leukemia and Beyond

**DOI:** 10.3389/fgene.2020.00598

**Published:** 2020-06-12

**Authors:** Peiqi Wang, Yun Deng, Xinyu Yan, Jianhui Zhu, Yuanyuan Yin, Yang Shu, Ding Bai, Shouyue Zhang, Heng Xu, Xiaoxi Lu

**Affiliations:** ^1^Department of Pediatric Hematology/Oncology, West China Second University Hospital, Sichuan University, Chengdu, China; ^2^State Key Laboratory of Oral Diseases, West China Hospital of Stomatology, Sichuan University, Chengdu, China; ^3^State Key Laboratory of Biotherapy, West China Hospital, Sichuan University and Collaborative Innovation Center, Chengdu, China; ^4^Department of Laboratory Medicine/Research Center of Clinical Laboratory Medicine, West China Hospital, Sichuan University, Chengdu, China; ^5^Precision Medicine Center, State Key Laboratory of Biotherapy and Precision Medicine, Key Laboratory of Sichuan Province, West China Hospital, Sichuan University and Collaborative Innovation Center, Chengdu, China

**Keywords:** ARID5B, acute lymphoblastic leukemia, susceptibility, single nucleotide polymorphism, chemotherapy

## Abstract

Acute lymphoblastic leukemia (ALL) is the most common malignancy in children with distinct characteristics among different subtypes. Although the etiology of ALL has not been fully unveiled, initiation of ALL has been demonstrated to partly depend on genetic factors. As indicated by several genome wide association studies (GWASs) and candidate gene analyses, ARID5B, a member of AT-rich interactive domain (ARID) protein family, is associated with the occurrence and prognosis of ALL. However, the mechanisms by which *ARID5B* genotype impact on the susceptibility and treatment outcome remain vague. In this review, we outline developments in the understanding of *ARID5B* in the susceptibility of ALL and its therapeutic perspectives, and summarize the underlying mechanisms based on the limited functional studies, hoping to illustrate the possible mechanisms of ARID5B impact and highlight the potential treatment regimens.

## Introduction

**FIGURE 1:**
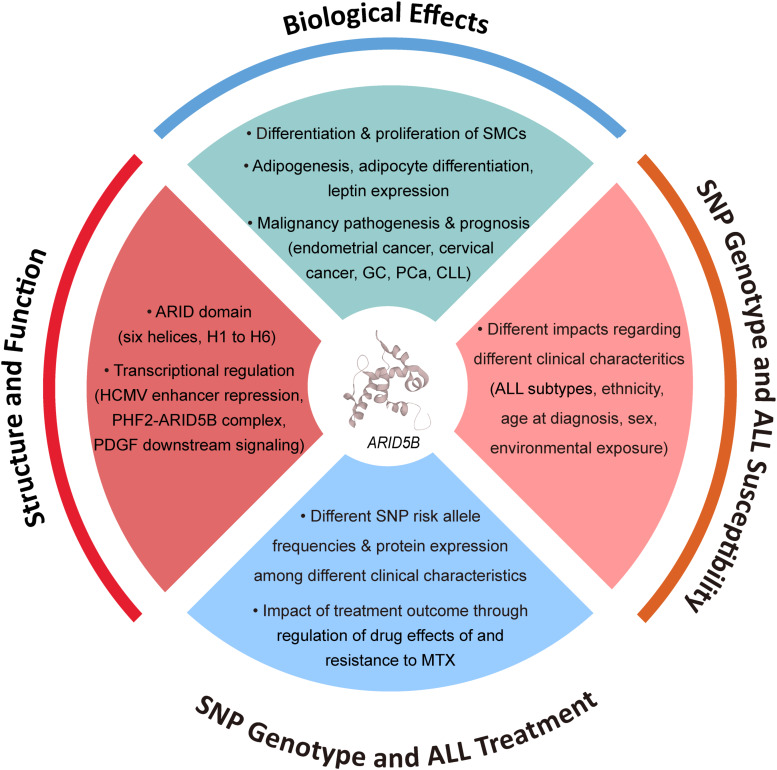
Factors associated with function of ARID5B and *ARID5B* SNPs.

Acute lymphoblastic leukemia (ALL), the most common malignancy in children, is a heterogeneous disease with subtypes that differ markedly in their cellular and molecular characteristics (Chen et al., 1997; Brisson et al., 2015). Although advances in perception of the pathobiology of ALL have led to risk-targeted therapeutics and increased long-term survival rates, the etiology of pediatric ALL remains poorly understood. Initiation of leukemogenesis occurs during fetal life or in early infancy and is likely to be caused by multiple environmental and genetic factors (Greaves, 2002; Chen et al., 2008). The assertion that ALL may have a genetic basis has long been pursued through genome wide association studies (GWASs) and association studies based on candidate genes. Genes involved in xenobiotic metabolism, oxidative stress response, DNA repair, folate metabolism and cell-cycle regulation have been associated with ALL. Among them, strong associations between variants at 10q21.2 (*ARID5B*) and childhood ALL risk have been repeatedly suggested (Figure 1).

ARID5B, also named as MRF2 (modulator recognition factor 2) or DESRT, belongs to the AT-rich interactive domain (ARID) protein family, members of which serve as epigenetic regulators by binding with specific or unspecific AT-rich sequences of genomic DNA, and interact with their partners to modulate chromatin structures (Herrscher et al., 1995; Gregory et al., 1996). Further studies have demonstrated that members of ARID family play a part in cell growth and differentiation as transcriptional regulators (Wilsker et al., 2002). Therefore, it is believed that dysfunctions of these genes may facilitate tumorigenesis, which has been proved by high-throughput screenings for inherited predispositions or tumor genomic mutations. ARID5B is essential for development of hematopoietic cells. Multiple single nucleotide polymorphisms (SNPs) in *ARID5B* gene have been reported as susceptibility markers for ALL in ethnically diverse populations (Papaemmanuil et al., 2009; Treviño et al., 2009). It is noticeable that heterogeneity of such association was noticed in patients with different characteristics including ALL subtype, ethnicity, age at diagnosis, etc. Additionally, risk alleles of *ARID5B* SNP genotypes as well as down-regulation of ARID5B have also been considered to be related to leukemia relapse.

In this review, we aim to focus on the association of *ARID5B* SNPs with ALL susceptibility and its therapeutic perspectives. Moreover, we will also demonstrate the molecular activities of this gene based on the limited functional studies, to illustrate the possible mechanisms of ARID5B impact on ALL and search for a more valid treatment regimen concerning ARID5B.

## Association of *ARID5B* With Acute Lymphoblastic Leukemia

ALL is one of the leading causes of disease-induced death in children around the world (Brisson et al., 2015; Chen et al., 1997). In the last few decades, the treatment outcome of childhood ALL has been largely improved with the development of antileukemic agents and risk-adapted therapy, whereas about 15% of the patients still suffer relapse and low curation rate afterward (Karathanasis et al., 2009). Therefore, it is of great importance to unveil the underlying mechanism of this aggressive cancer.

### Impact of *ARID5B* SNP Genotype on ALL Susceptibility

Both genetic background and environment exposure to leukemogenic agents would affect the onset and development of ALL (Greaves, 2002; Chen et al., 2008). The early onset and familial aggregation of the disease suggest a strong inherited genetic basis of ALL susceptibility (Hemminki and Jiang, 2002; Greaves et al., 2003). Indeed, functional germline mutations of some cancer-related genes have been found in familial ALL (e.g., *PAX5* and *ETV6*) (Shah et al., 2013; Noetzli et al., 2015) or enriched in sporadic cases (e.g., *ETV6* and *CDKN2A*) (Moriyama et al., 2015; Xu et al., 2015), accounting for a small proportion of ALL patients. Importantly, unbiased GWAS provided the opportunity to find common genetic basis of diseases (McCarthy et al., 2008). In 2009, two independent GWASs studied inherited predispositions to ALL susceptibility in Caucasians, identifying 10q21.2-*ARID5B* as one of the locus with strongest association signals (Papaemmanuil et al., 2009; Treviño et al., 2009). A total of five SNPs (i.e., rs7073837, rs10740055, rs7089424, rs10821936, rs10994982) in *ARID5B* were associated with childhood B-ALL in these two GWASs, among which rs7089424 showed the strongest signal (Papaemmanuil et al., 2009), and it was in high linkage disequilibrium (LD) with rs10821936 (Treviño et al., 2009). Noticeably, all the aforementioned SNPs mapped to or exhibited high LD with intron 3 of the gene *ARID5B*, despite that how the region increases ALL susceptibility remains unknown. Since then, a series of replication studies in independent ALL patient cohorts have focused on the association of reported *ARID5B* SNPs with ALL susceptibility, exhibiting positive results among different ethnic groups (Table 1).

**TABLE 1:** *ARID5B* SNPs with ALL susceptibility among different ethnic groups.

SNP	ALL subtype	Population characteristics	OR	95% CI	Cases	Controls	*P* value	Study type	Country	PMID	References
rs10821936	Overall	European	1.91	1.60–2.20	317	17958	1.40 × 10^–15^	GWAS	United States	19684603	Treviño et al., 2009
rs10821936	B-hyperdiploid ALL	European	2.17	1.50–3.10	124	17958	1.62 × 10^–5^	GWAS	United States	19684603	Treviño et al., 2009
rs10821936	Overall	European American	1.88	1.68–2.10	972	1386	6.9 × 10^–30^	GWAS	United States	23512250	Xu et al., 2013
rs10821936	Overall	African American	1.52	1.14–2.02	89	1363	0.004	GWAS	United States	23512250	Xu et al., 2013
rs10821936	Overall	Hispanic American	1.95	1.60–2.38	305	1008	3.78 × 10^–11^	GWAS	United States	23512250	Xu et al., 2013
rs10821936	Overall	Multi-ethnics	1.86	1.71–2.03	1605	6661	5.88 × 10^–46^	GWAS	United States	23512250	Xu et al., 2013
rs10821936	Overall	Whites	2.13	1.77–2.58	978	1046	2.19 × 10^–15^	Candidate gene	China	22291082	Xu et al., 2012
rs10821936	Overall	Hispanics	1.92	1.50–2.45	330	541	2.14 × 10^–7^	Candidate gene	China	22291082	Xu et al., 2012
rs10821936	Overall	Blacks	2.09	1.31–3.30	93	112	0.0015	GWAS	United States	20054350	Yang et al., 2010
rs10821936	Overall	Non-Hispanic Whites	1.91	1.60–2.20	317	17958	1.40 × 10^–15^	GWAS	United States	20054350	Yang et al., 2010
rs10821936	B-hyperdiploid ALL	Blacks	6.62	2.02–21.90	16	112	0.0021	GWAS	United States	20054350	Yang et al., 2010
rs10821936	B-hyperdiploid ALL	Non-Hispanic Whites	4.63	1.67–2.82	108	17958	1.30 × 10^–10^	GWAS	United States	20054350	Yang et al., 2010
rs10821936	*ETV6-RUNX1* ALL	Blacks	2.01	0.82–4.96	23	112	0.13	GWAS	United States	20054350	Yang et al., 2010
rs10821936	*ETV6-RUNX1* ALL	Non-Hispanic Whites	1.78	0.92–3.64	45	17958	0.09	GWAS	United States	20054350	Yang et al., 2010
rs10821936	Overall	Chinese	1.81	1.42–2.30	–	–	<0.0001	Candidate gene	China	23608171	Wang et al., 2013
rs10821936	B-cell ALL	Latvian population	1.67	1.10–2.67	–	–	0.028	Candidate gene	Latvia	27279837	Prasad et al., 2010
rs10821936	Overall	Hungarian	1.43	1.20–1.71	543	529	7.31 × 10^–5^	Candidate gene	Hungary	23021489	Lautner-Csorba et al., 2012
rs10821936	B-cell ALL	Hungarian	1.53	1.26–1.85	390	529	1.95 × 10^–5^	Candidate gene	Hungary	23021489	Lautner-Csorba et al., 2012
rs10821936	B-cell ALL	European	2.18	1.48–3.20	129	99	1.70 × 10^–7^	Candidate gene	Canada	20460642	Healy et al., 2010
rs10821936	B-cell ALL	Indian	0.67	0.47–0.94	162	150	0.019	Candidate gene	India	27644650	Bhandari et al., 2016
rs10821936	Overall	Multi-ethnics	2.57	1.74–3.79	160	43	9 × 10^–7^	Candidate gene	United States	23692655	Linabery et al., 2013
rs10821936	B-hyperdiploid ALL	Multi-ethnics	8.42	4.11–17.25	30	43	1 × 10^–9^	Candidate gene	United States	23692655	Linabery et al., 2013
rs10821936	B-cell ALL	Spanish	1.84	1.23–2.75	219	397	4.5 × 10^–7^	Candidate gene	Spain	24013273	Gutierrez-Camino et al., 2013
rs10821936	*MLL*-germline ALL	Non-hispanic Europeans	7.20	2.50–20.60	11	43	0.0002	Candidate gene	United States	22422485	Ross et al., 2013
rs10821936	*MLL*-germline ALL	Whites and Non-whites	2.77	1.40–5.47	45	273	–	Candidate gene	Spain	24564228	Emerenciano et al., 2014
rs10821936	*MLL*-r ALL	Whites and Non-whites	3.04	1.61–4.72	67	273	–	Candidate gene	Spain	24564228	Emerenciano et al., 2014
rs7089424	Overall	European	1.65	1.54–1.76	907	2,398	6.70 × 10^–19^	GWAS	United Kingdom	19684604	Papaemmanuil et al., 2009
rs7089424	Overall	Tunisian population	0.49	0.31–0.79	58	150	0.0022	Candidate gene	Thailand	27184773	Gharbi et al., 2016
rs7089424	Overall	Mexican	2.00	1.60–2.50	285	476	–	Candidate gene	Mexico	28476190	Bekker-Mendez et al., 2016
rs7089424	Overall	Yemeni	2.19	1.08–4.45	136	153	0.02	Candidate gene	Malaysia	28381164	Al-Absi et al., 2017
rs7089424	Overall	Hispanics	1.98	1.59–2.48	300	406	1 × 10^–9^	Candidate gene	United States	23836053	Chokkalingam et al., 2013
rs7089424	Overall	Non-hispanic Whites	1.84	1.43–2.37	225	369	2.2 × 10^–6^	Candidate gene	United States	23836053	Chokkalingam et al., 2013
rs7089424	Overall	Polish	1.94	1.26–3.00	398	731	0.003	Candidate gene	Poland	21889209	Pastorczak et al., 2011
rs7089424	Precursor B-cell ALL	European	1.80	1.62–2.00	1384	1877	5.9 × 10^–28^	Candidate gene	Germany	20042726	Prasad et al., 2010
rs10994982	B-hyperdiploid ALL	European	1.72	1.20–2.40			0.003	GWAS	United States	19684603	Treviño et al., 2009
rs10994982	*MLL*-germline ALL	Whites and Non-whites	2.97	1.08–8.12	34	377	–	Candidate gene	Spain	24564228	Emerenciano et al., 2014
rs10994982	*MLL*-r ALL	Whites and Non-whites	1.50	0.70–3.22	72	377	–	Candidate gene	Spain	24564228	Emerenciano et al., 2014
rs10821938	Precursor B-cell ALL	Thai population	0.73	0.55–0.97	190	182	0.03	Candidate gene	Thailand	20919861	Vijayakrishnan et al., 2010

Several meta-analyses systematically reviewing the independent association studies also highlighted the strong impact of *ARID5B* SNPs on the enhanced risk of childhood ALL (Guo et al., 2014; Zeng et al., 2014; Yang et al., 2019). Collectively, it is reasonable to state that unequivocal evidence has been provided for inherited genetic background of ALL pathogenesis with *ARID5B* serving as one of the most critical loci. Meanwhile, these studies also exhibited the impact of clinical characteristics, such as subtype, ethnicity, age at diagnosis, on risk allele frequencies (RAF) and odds ratio (OR) of ALL.

#### Impact of *ARID5B* SNP Genotype on ALL Susceptibility in Different Genetic Subtypes

ALL is composed of two main immunophenotypes that are identified by distinctive hematopoietic lineage markers: B-cell ALL (B-ALL) and T-cell ALL (T-ALL). Pediatric B-ALL is diagnosed in up to 85% of ALL cases while T-ALL comprises the remaining 15% (Pui et al., 2015). Moreover, multiple epidemiological and molecular studies have already demonstrated a crucial role of abnormalities in chromosome number as well as structural rearrangements in ALL. Among them, *ETV6-RUNX1* gene fusion is the most frequently occurring single genetic abnormality in pediatric leukemia. It is a prenatal event which generates persistent preleukemic clones that may postnatally convert to ALL after acquisition of acquisition of necessary secondary genetic lesions (Sundaresh and Williams, 2017). The t(1;19)(q23;p13) which results in *TCF3/PBX1* chimeric gene is also frequently observed in B-ALL with an overall frequency of 6% in both adult and pediatric populations (Tirado et al., 2015). It encodes a transcription factor bearing the transactivation domain of TCF3 and the DNA-binding domain of PBX1, which facilitates the activation or repression of genes (Hajingabo et al., 2014). In addition, the *Mixed Lineage Leukemia* (*MLL*) gene at 11q23 are found in 10 and 5% of adult and children ALL cases, respectively. Up to 80% of ALL arising in infants less than 1 year of age are characterized by *MLL*-rearrangements (*MLL*-r). *MLL*-r ALL represents a distinct leukemia with hyperleukocytosis, aggressive behavior with early relapse, relatively high incidence of central nervous system involvement and its epigenetically acting mechanism has been widely studied (Williams et al., 2019; El Chaer et al., 2020).

It should be remarked that the biologically different subtypes of ALL suggest different etiologies (Greaves, 2006), and thus risk variants are also likely to have different effects on ALL susceptibility depending on cell lineage and phenotype. For instance, *ARID5B* SNP rs10821936 is associated with a significantly increased risk of overall ALL. While this association steadily persists among B-ALL cases across different ethnic groups (Healy et al., 2010; Lautner-Csorba et al., 2012; Xu et al., 2012; Chokkalingam et al., 2013; Linabery et al., 2013; Bhandari et al., 2016), it is null among Caucasian children and increases among black children when it comes to T-ALL (Lautner-Csorba et al., 2012; Linabery et al., 2013; Yang et al., 2010). Within B-ALL, *ARID5B* SNPs show strongest impact on ALL susceptibility in terms of B-hyperdiploid ALL (Yang et al., 2010; Lautner-Csorba et al., 2012; Chokkalingam et al., 2013; Linabery et al., 2013). Several studies have confirmed more common appearance of rs10821936 C allele in hyperdiploid ALL than in ALL with the *TCF3-PBX1* or *ETV6-RUNX1* fusion genes in either whites or non-white Hispanics (Papaemmanuil et al., 2009; Treviño et al., 2009). Specifically, we further confirmed the results in 2012, indicating RAF of rs10821936 C allele as 53.1, 47.6, and 40.4% in hyperdiploid ALL, *TCF-PBX1* ALL, and *ETV6-RUNX1* ALL, respectively. As to *MLL*-r ALL enriched in infant ALL patients, a study in 2012 conducted by Ross et al. showed that *MLL*-r leukemia had much lower RAF of rs10821936 than those without such translocation, revealing a significant risk with rs10821936 among ALL/*MLL*-germline cases only (Ross et al., 2013). However, in 2014, Emerenciano et al. came to the opposite conclusion, stating that *ARID5B* rs10821936 conferred increased risk to both *MLL*-germline and *MLL*-r leukemia in whites as well as non-whites (Emerenciano et al., 2014). Since the aforementioned two researches both focused on early age leukemia (EAL), we assume that ethnicity, sample sizes, and analytic methods may contribute to the different conclusion. Moreover, the ORs relating *ARID5B* rs10821936 genotype to the other subtype of B-ALL was shown to be moderate in either white or black patients with *ETV6-RUNX1* fusion (Yang et al., 2010). Meanwhile, no systematic examination has been reported on ALL patients with *BCR-ABL* fusion. Although not specifically mentioned in Ph-like ALL, it can be speculated that *ARID5B* SNPs have much lower effect on this subtype in either children or adults, because they didn’t reach the genome-wide significance.

#### Impact of *ARID5B* SNP Genotype on ALL Susceptibility According to Ethnicity

As researchers suggested, ethnicity is related to ALL subtypes as well as susceptibility and prognosis. Studies have noticed higher ALL incidence in Hispanics (Linabery and Ross, 2008; Chow et al., 2010) and better prognosis in Caucasians (Bhatia et al., 2002; Kadan-Lottick et al., 2003). Approximately 85% of white, 87% of Hispanic, 81% of Asian, and 75% of black children are diagnosed with B-ALL and the remaining proportion of child ALL patients in each ethnicity are diagnosed with T-ALL (Bhatia et al., 2002; Kadan-Lottick et al., 2003; Pui et al., 2004; Yang et al., 2010), which has lower survival than B-ALL (Hunger et al., 2012). Impacts of risk factors on ALL susceptibility are likely to differ in line with ethnic groups, and although *ARID5B* SNPs generally show similar association with ALL susceptibility, differences still exist. Despite that non-European populations were undeniably underrepresented for leukemia in earlier genetic studies, recent researchers have focused more on diverse races (Rosenberg et al., 2010; Bustamante et al., 2011). As mentioned above, the association between rs10821936 and T-ALL is null among Caucasian children (Lautner-Csorba et al., 2012; Linabery et al., 2013), and yet increases among black children (Yang et al., 2010). To further understand the effect of *ARID5B* SNPs on ALL risk among different ethnicities, our research group conducted a large GWAS for ALL susceptibility in ethnically diverse populations and indicated the consistent association of *ARID5B* with ALL susceptibility across all ethnicities (Xu et al., 2013). Both ORs and RAF of rs10821936 for ALL susceptibility increased in the order of Africans (RAF = 33%, OR = 1.52), Caucasians (RAF = 48%, OR = 1.88) and Hispanics (RAF = 63%, OR = 1.95) (Xu et al., 2012, 2013), which is in parallel with ALL incidence among ethnic/racial groups (incidence rate: Africans < Caucasians < Hispanics) (Linabery and Ross, 2008; Dores et al., 2012). A replication study also supported this assumption in East Asian patients and Caucasians, who had similar ALL incidence as well as RAF and OR for rs10821936 (Wang et al., 2013). Interestingly, a study in 2016 regarding Indian population indicated the association of *ARID5B* rs10821936 with decreased B-lineage ALL susceptibility in Indian children (OR = 0.67, 95% CI = 0.47–0.94, *p* = 0.019), further confirming the idea that variants in SNPs may confer different risk of ALL within different populations (Bhandari et al., 2016).

#### Impact of *ARID5B* SNP Genotype on ALL Susceptibility Regarding Age at Diagnosis, Sex, and Environmental Exposure

The incidence of ALL is highly related to age with the majority of cases occurring in children aged 2–5 years (Greaves, 2006). Hitherto, age at diagnosis has been proved to connect to the etiologic heterogeneity between subtypes of ALL, serving as a proxy of some cytogenomic subtypes (Greaves, 2006). It has been reported, for example, 80% of infant ALL cases, diagnosed in children less than 1 year of age, have an *MLL*-r, while approximately 35% of cases with B-ALL aged 1–9 years are diagnosed with the hyperdiploid subtype and another 30% are diagnosed with *ETV6-RUNX1* fusions (Hunger and Mullighan, 2015). It was shown that although the ORs for some other gene SNPs were similar for pediatric and adult ALL, ORs for *ARID5B* rs7089424 and rs10821936 got much lower in adult ALL. This may be partly due to the lower frequency of hyperdiploid B-ALL in adults (Burmeister et al., 2014). Our previous GWAS of susceptibility to ALL in adolescents and young adults has also indicated that *ARID5B* SNPs lost their genome-wide significance in patients in such age groups (Perez-Andreu et al., 2015). Moreover, we have also proved that as age at diagnosis increased, the trend in OR for rs10821936 decreased among ALL (all subtypes), hyperploid ALL, and *ETV-RUNX1* ALL in an ethnic independent manner (Xu et al., 2013).

A putative gender-specific effect of *ARID5B* SNPs on ALL risk has been reported by a multivariate haplotype analysis among Europeans. The risk haplotype AACCG was associated with a near 2-fold increase in B-cell ALL susceptibility (OR = 1.93, 95% CI = 1.47–2.53, *p* = 7.6 × 10^–7^) in male subgroup only (Healy et al., 2010). However, controversial results have been suggested in a study concerning French children, showing that associations with *ARID5B* SNPs were slightly more marked in females than in males (Orsi et al., 2012). Intriguingly enough, a study by Linabery et al., indicated that allele frequency of rs10821936 did not differ between males (RAF = 42%, OR = 1.38) and females (RAF = 47%, OR = 1.58) (Linabery et al., 2013). Similarly, our large-scale study came to the same conclusion with C allele frequency at rs10821936 48.6 and 47.2% among white males and females, repectively, and 63.4 and 61.4% among Hispanic males and females, respectively (Xu et al., 2013).

Despite that environmental exposures to parents before and after conception have always been believed to be associated with risk alleles, no independent study has supported this conjecture in *ARID5B* SNPs. No interaction was apparent for *ARID5B* variant rs7089424 with any of these exposures paternal smoking, maternal folate and alcohol use (each before conception) (Evans et al., 2014). Neither have significant differences been observed in the distribution of *ARID5B* genotypes across stratum of birth weight, or maternal age (Linabery et al., 2013).

### Association of *ARID5B* SNP Genotype With ALL Treatment Outcome

Around 15% of ALL patients suffer relapse after treatment, and minimal residual disease (MRD) is considered as one of the strongest prognostic factors. Remarkably, the risk alleles of *ARID5B* SNPs for ALL susceptibility are related to poorer treatment outcome. Most of the SNPs associated with ALL relapse were linked with MRD status at the end of remission induction therapy, and some stayed prognostic even after adjusting for MRD (Bhatia et al., 2002; Kadan-Lottick et al., 2003; Xu et al., 2012). Moreover, the risk allele frequencies of these *ARID5B* SNPs varied among ethnicities and were enriched in patients with younger age at diagnosis, which, on the other hand, partially explains the impact of ethnicity and age at diagnosis on ALL treatment outcomes (Xu et al., 2012, 2013).

Although no specific mechanism for such association has been addressed, we assume that ARID5B-related treatment outcome could be related to drug effects of and resistance to antileukemic drugs. Our group recently confirmed that ARID5B expression varied substantially by ALL subtype, with the highest level being observed in hyperdiploid ALL. Lower ARID5B expression at diagnosis was associated with the risk of ALL relapse, and further reduction was noted at ALL relapse. We indicated a determinant role of ARID5B to control drug sensitivity of antimetabolite drugs including mercaptopurine and methotrexate (MTX). ARID5B knockdown led to resistance specific to antimetabolite drugs in part through p21-mediated cell-cycle arrest (Xu et al., 2020). The results supplemented the existing data concerning the relationship of ARID5B and MTX, an anticancer agent widely used in the risk-adjusted therapy of childhood ALL (Moricke et al., 2008). Large interindividual variability of MTX response has been observed even for patients with the same protocol (Mikkelsen et al., 2011; Csordas et al., 2013). Patients with *ARID5B* SNP risk alleles were proven to have greater intracellular accumulation of MTX polyglutamates (MTXPGs), which mainly determine the cytotoxicity and antileukemic effects of MTX, especially in individuals with hyperdipoid B-ALL (Masson et al., 1996; Treviño et al., 2009), concomitant with the better response of B-ALL to MTX chemotherapy (Kager et al., 2005; Treviño et al., 2009; Xu et al., 2012). Additionally, *ARID5B* SNPs also exhibited significant associations with the serum MTX and 7-OH-MTX (a metabolite of MTX converted in hepatocytes), as well as the development of hypoproteinaemia (Csordas et al., 2014). More studies are needed to investigate the way risk alleles of *ARID5B* SNPs impact on MTX metabolism, and further examinations of *ARID5B* variation in the context of different ALL treatment regimens are warranted to refine its value as a prognostic marker.

## The Structure and Function of ARID5B

The human ARID family contains 15 members, which can be divided into 7 subgroups in terms of the similarity of their sequences and protein structures of ARID domain, whose consensus sequence extends across ∼100 amino acids (Wilsker et al., 2002; Patsialou et al., 2005). All these proteins have been implicated in transcriptional regulation, and are considered to be involved in a variety of biological processes (e.g., stem cell development, cell cycle control) through modifying chromatin structure and positively/negatively regulating transcription of the downstream targets in a tissue-specific manner (Zhang et al., 2019). While the basic structure appears to be a series of six α-helices separated by strands, loops, or turns, the region may extend to an additional helix at either or both ends of the basic six. Besides, despite that the shared sequence is highly conserved, the ARID region itself shows more diversity in structure and function (Iwahara and Clubb, 1999; Patsialou et al., 2005). For instance, several ARID proteins can only non-specifically bind to AT-rich DNA domain (e.g., ARID1A) (Dallas et al., 2000), while others (e.g., ARID5B) can specifically bind to the core sequence AATA(C/T) for high affinity (Whitson et al., 1999).

**FIGURE 2:**
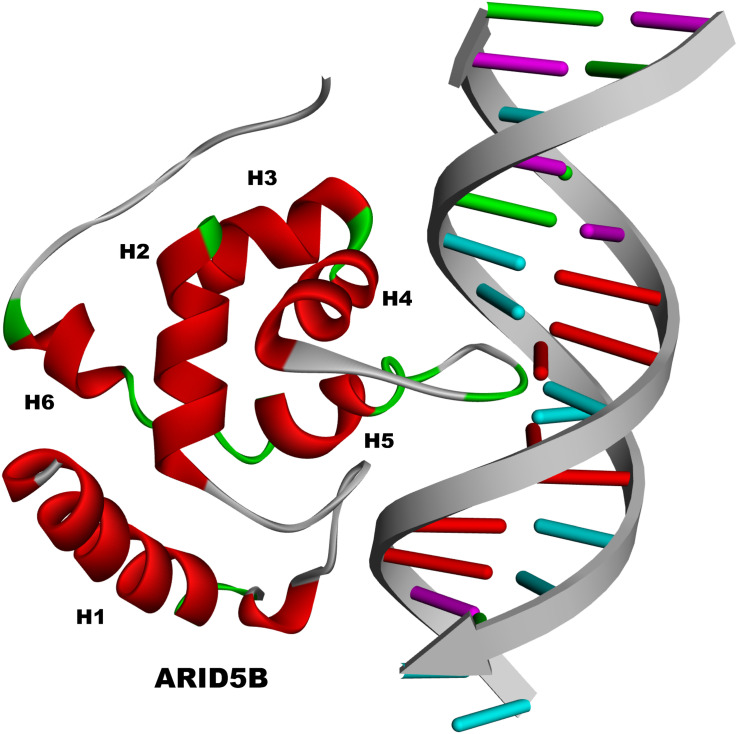
The structure of *ARID5B*.

The ARID domain of ARID5B contains six helices (H1 to H6) just as the basic structure does, with a loop between H1 and H2 (Figure 2). H5 and its preceding turn serve for DNA contact and sequence recognition through interacting with the major groove of DNA, whereas other residues contact the minor groove or phosphate backbone. Meanwhile, the loop between H1 and H2 of ARID5B is also believed to be involved in the contact with the minor groove. The flexible COOH terminus of ARID5B may form additional important contacts with the minor groove or phosphate backbone (Zhu et al., 2001). Moreover, well-defined three-dimensional structure of ARID5B has been obtained by using distance constraints derived from paramagnetic line-broadening effects and docking calculations based on nuclear magnetic resonance (NMR) chemical shift perturbation. The three-dimensional structure revealed that ARID5B cannot only interact with DNA through both the major and minor grooves, but also share structural homology to DNA replication and repair nucleases and polymerases (Cai et al., 2007). In addition, ARID5B is also termed as MRF2 and has two isoforms of MRF2α (3.0 kb) and MRF2β (3.7 kb), which differ in the N-terminus but share the ARID DNA binding domain encoded by exons III-VI (Yamakawa et al., 2008).

Noteworthily, the reason for the name “MRF2” lies in that it was firstly cloned by virtue of its ability to bind to similar sequences in the transcriptional modulator of the human cytomegalovirus (HCMV) major immediate-early promoter, serving as a repressor of the HCMV enhancer in undifferentiated human teratocarcinoma cell line Tera-2 and human monocytic cell line THP-1, which is similar with MRF1 (Huang et al., 1996). Although MRF1 and MRF2/ARID5B have highly homologous regions of 108 amino acids with 80 identical residues and 13 conservative substitutions (Yuan et al., 1998), they are not related outside of the ARID domain. Moreover, retinoic acid-induced differentiation in Tera-2 and THP-1 cells was shown to result in reduced ARID5B DNA binding activity and activation of the aforementioned enhancer, indicating that the DNA binding activity of MRF2/ARID5B may be regulated by its binding to the retinoic acid receptor (Huang et al., 1996).

More recent studies suggest that ARID5B plays a part in transcription regulation through histone modification by forming the histone demethylase complex with plant homeodomain finger protein 2 (PHF2), a jmjC histone lysine demethylase, through an N-terminal region (Whetstine et al., 2006; Christensen et al., 2007; Klose et al., 2007; Shi and Whetstine, 2007). PHF2 becomes enzymatically active through PKA-induced phosphorylation and demethylates ARID5B at Lys336 before forming the complex. The formed complex then anchors on the target gene promoters, where PHF2 mediates demethylation of dimethylated Lys 9 on histone H3 (H3K9Me2), the repressive histone methylation mark of gene transcription whose demethylation of promoters permits target gene transcription. In this way, the PHF2-ARID5B complex induces transcription activation of target genes (Baba et al., 2011). This phenomenon has mainly been identified in adipogenesis and liver development. It was proved that PHF2-ARID5B acted as a co-activator of hepatocyte nuclear factor 4α (HNF4α), the central activator on promoters of gluconeogenic enzymes including Pepck and G6Pase in liver cells (Yoon et al., 2001; Rhee et al., 2003; Koo et al., 2005), and thus took part in the maintenance of glucose homeostasis. Moreover, during the process of chondrogenesis, the PHF2-ARID5B complex has been reported to, with Sox9 physically attached to, modulated H3K9me2 levels of chondrocyte marker gene promoters and subsequently allowed Sox9-induced chondrogenic gene transcription (Hata et al., 2013).

Besides being regulated by retinoic acid receptor mentioned above, ARID5B has also been stated as one of the immediate early genes (IEGs) induced shortly after activation of platelet-derived growth factor (PDGF) signaling. It was demonstrated that ARID5B performed in a network controlling specific PDGF signaling downstream process, and that mice with homozygous loss of ARID5B exhibited reduced growth rates, kidney defects, malformations of skeletal structures and decreased cell migration of embryonic fibroblasts (Schmahl et al., 2007).

## The Biological Effects of ARID5B

ARID5B was reported to be the regulator in the differentiation and proliferation of smooth muscle cells (SMCs). In the process of a pluripotent neural crest cell line (MONC-1) differentiating into SMCs, both of ARID5/MRF2 isoforms (MRF2α and MRF2β) were significantly induced. And the overexpression of MRF2α and MRF2β increased the expression of smooth muscle marker genes and retarded proliferation of SMCs (Watanabe et al., 2002).

Moreover, functions of ARID5B in lipid accumulation have also been revealed in several studies. Mice with targeted disruption of ARID5B were proved to be lean with significantly lower levels of adipose tissue and body fat (Whitson et al., 2003). Correspondingly, ARID5B knockdown of mouse fibroblasts and 3T3-L1 preadipocytes resulted in suppression of lipid accumulation and a significant decrease in the expression of important transcription factors of adipogenesis such as C/EBPα and PPARγ (Yamakawa et al., 2008). Since these transcription factors also contribute to the function of the maintenance of adipocyte function, studies were conducted to examine the effects of ARID5B knockdown on triglyceride metabolism. siRNA targeted to ARID5B was shown to activate synthesis of both lipolysis and triglyceride, and accelerate fatty acid recycling, indicating the negative role of ARID5B in triglyceride metabolism (Yamakawa et al., 2010). Furthermore, in preadipocytes and mature adipocytes, the expression of ARID5B gene was up-regulated during the differentiation of 3T3-L1 derived adipocytes and in response to different stimuli, while down-regulation of ARID5B increased the expression of leptin mRNA (Dong et al., 2008). These results demonstrated that ARID5B may act as a significant regulator of adipogenesis, adipocyte differentiation and leptin expression. Claussnitzer et al. (2015) demonstrated that *FTO* obesity variants disrupted ARID5B binding in the risk haplotype, leading to a loss of repression and increases in IRX3 and IRX5, the development regulators implementing long-range (1.2 Mb) genetic control in primary preadipocytes. In non-risk-allele carriers, overexpression of ARID5B negatively regulated IRX3 and IRX5 while its knockdown enhanced IRX3 and IRX5 expression (Claussnitzer et al., 2015), which is consistent with the aforementioned phenomenon. On the other hand, however, ARID5B was positively correlated with expression of IRX3 in breast cancer cells surviving metabolic challenge (Singh et al., 2016). Together, the results suggested that ARID5B may play a controversial role in triglyceride metabolism, serving as a repressor or as an activator depending upon cellular context.

More importantly, several studies aiming at different disease settings have shown insights into the role of ARID5B in malignancy pathogenesis and prognosis. In 2013, The cancer Genome Atlas (TCGA) research network provided a novel finding for endometrial cancer, suggesting ARID5B was more frequently mutated in microsatellite instability (MSI) (23.1%), than in either microsatellite stable (MSS) endometrioid (5.6%) or high somatic copy number alteration (SCNA) serous tumors (0%) (Cancer Genome, Atlas Research et al., 2013). A recent study comparing genetic-and-epigenetic cell cycle networks (GECNs) of embryonic stem cells (ESCs) and cervical cancer cells showed that *ARID5B* was one of the eight cell cycle genes whose methylation patterns significantly differ between ESCs and HeLa cells (Li and Chen, 2016). The methylation of *ARID5B* could focus on the specific cell cycle genes of cervical cancer cells and would have minimal side-effects on the shared core genes and thus provide greater therapeutic benefits (Berger and Iyengar, 2009; Zhao and Iyengar, 2012). Other than this, ARID5B has also been reported to associate with the ontogeny and evolution of gastric cancer (GC) and prostate cancer (PCa). It serves as a target gene of TEA domain 4 (TEAD4), whose dysregulation at epigenetic, transcriptional and posttranscriptional levels would contribute to the development of GC (Lim et al., 2014). The dysregulation of ARID5B has also been evidenced to play a part in the regulatory pathways of PCa by comparing the proteins of PCa and benign prostate hyperplasia (BPH) tissue (Davalieva et al., 2015). Moreover, ARID5B has been revealed to be involved in fludarabine-refractory of chronic lymphocytic leukemia (CLL) patients. The data showed a modest but significant increase in F-ara-A (the nucleoside pro-drug of fludarabine) IC50 attributed to the overexpression of ARID5B, partly through regulation of mitogen-activated protein kinases (MAPK) pathway, whose inhibition results in fludarabine resistance in CLL (Pandzic et al., 2016).

## Potential Mechanisms for the Impact of *ARID5B* SNP Genotype

Although how exactly ARID5B is connected to ALL remains unknown, it is safe to assume that it may be involved in epigenetic regulation of gene expression in hematopoietic stem cells and early lymphoid progenitors like other AT-rich DNA-binding proteins (Webb et al., 2011; Yokota and Kanakura, 2014). A study in 2001 stated that mice with a targeted mutation in the ARID domain of *Arid5b* exhibited transient reductions in B-lymphocyte accounts in the bone marrow and secondary lymphoid organs, as well as reduced proportions of lymphocyte progenitors in those organs (Lahoud et al., 2001). Our Vav-1 specific *Arid5b* overexpression (AOE) mouse model also presented a significant reduction in the proportion of all bone marrow B cell populations and a loss of functional pre B lymphoid progenitor, indicating a crucial tole of *Arid5b* in B lymphopoiesis and erythropoiesis (Goodings-Harris et al., 2018). Moreover, *ARID5B* mRNA expression was upregulated in hematologic malignancies such as acute promyelocytic leukemia (Chang et al., 2008) and acute megakaryoblastic leukemia (Bourquin et al., 2006). Leong et al. (2017) demonstrated that *ARID5B* was abnormally activated by TAL1 complex in T-ALL and could positively regulate the TAL1-induced regulatory circuit and the oncogene *MYC*, thus reinforcing the oncogenic transcriptional program (Leong et al., 2017). Recently, they further suggested that in TAL1-positive T-ALL cases, ARID5B inducing enhancer associated long non-coding RNA (ARIEL), the enhancer RNA, was stimulated and played an oncogenic role in the process by activating *ARID5B* (Tan et al., 2019). As stated, *ARID5B* genotype is closely associated with All with specific gene fusions or genomic rearrangements. The heterozygous genotype in *ARID5B* rs10821936 was demonstrated to increase the risk of as *MLL-MLLT3/AF9* (Emerenciano et al., 2014). Another molecular study revealed novel *ARID5B-MLL* gene fusions in a case of infantile *MLL*-r ALL with complex karyotype (Hiwatari et al., 2017). Therefore, it is possible that the presence of a variant allele or aberrant expression of *ARID5B* may lead to transient abnormalities in immune cell development and distribution, and the process may be accompanied by differential expression or rearrangement of other transcript variants.

Since the risk variants either locate at or exhibit high LD with intron three of the gene *ARID5B*, they cannot serve as directly functional coding variants (Freedman et al., 2011). Hence it is reasonable to speculate that these alleles lie in the regulatory regions (e.g., enhancers and promoters) and impact the phenotype through affecting RNA splicing, transcription factor binding, promoter methylation, etc. as suggested in causal variants of other genes (Huang, 2015). Indeed, Besides Studd et al. (2017) identified a potential causal variant (rs7090445) for hyperdiploidy ALL risk at ARID5B locus which localized to intron three of ARID5B, and found its risk allele served to reduce enhancer activity of ARID5B in leukemic blasts (Studd et al., 2017). The study also suggested a correlation between rs7090445-C risk allele and disrupted RUNX3 binding, leading to the decrease in RUNX3-dependent ARID5B expression (Studd et al., 2017). It can be inferred that inherited genetic variations of ARID5B SNPs contribute to reduced ARID5B expression and blocking of normal lymphocyte development, and thus facilitate leukemic clonal expansion. In addition, as described above, risk alleles of multiple *ARID5B* SNPs are also associated with higher ALL relapse (Xu et al., 2012). We assume that the causal variant (if it is not rs70904450) may also facilitate ALL relapse through down-regulation of *ARID5B* expression, which is consistent with the fact that lower ARID5B expression are found in relapse blasts than their matched initial diagnostic blasts (Hogan et al., 2011). Interestingly though, as mentioned above, enhanced expression and activation of *ARID5B* have also been observed in multiple hematologic malignancies including T-ALL. The contradictory evidence could possibly be due to the difference in malignancy subtypes and the disorder of ARID5B expression might serve diverse roles in different stages of ALL.

## Conclusion

Encoded by *ARID5B* gene, ARID5B mainly serves as a transcriptional modulator, and regulates the expression of target genes by recruiting PHF2, the catalyzer for histone demethylation. It has been identified to be implicated in cell and tissue development and its aberrant expression is always connected with malignancy pathogenesis. A number of genetic studies have provided valid evidence for the association of germline variants at *ARID5B* loci with increased or decreased risk of developing childhood ALL. Of note, these *ARID5B* SNPs are specifically correlated with B-ALL and higher MTXPG level in B lymphocytes, providing a possible explanation for the better response of B-ALL to MTX. Germline variants also contribute to race differences in ALL incidence and recurrence probability (blacks < whites < Hispanics). However, the roles of age at diagnosis, gender and other clinical characteristics in these SNPs’ correlations with ALL risk remain unclear considering the contradictory results. Up to now, the mechanisms by which the risk alleles predispose to ALL and other diseases are still poorly understood. Although current researches have demonstrated *ARID5B*’s contribution to tumorigenesis and revealed part of the underlying mechanism, the studies were conducted in different disease background, and existing data present inconsistent results concerning the relationship of expression levels of *ARID5B* and ALL. Hence, we can only conjecture *ARID5B* in different malignancy initiation may not share universal mechanism. Also, in spite that several ARID5B SNPs have been suggested to contribute to ALL, how they influence ARID5B expression and differential expression of transcript variants remains little touched. Therefore, further investigation is awfully needed to focus on delving into the underlying mechanism of ARID5B SNPs in different neoplastic settings, facilitating the clarification of the role *ARID5B* plays in the etiology of leukemia and autoimmune diseases. As *ARID5B* SNPs are closely correlated to the onset and outcome of childhood ALL, the published findings warrant extensive genetic and functional studies to unravel the molecular mechanisms and evaluate the diagnostic and therapeutic significance of *ARID5B* for ALL.

## Author Contributions

PW, SZ, HX, and XL designed the review and made a retrieval strategy. PW, JZ, and YS collected and summarized current evidences and progress. PW, XY, and JZ drafted the review. YD and YY drafted the tables and figures. DB provided constructive advice during manuscript refinement. All the authors contributed to revision and finalization of the manuscript.

## Conflict of Interest

The authors declare that the research was conducted in the absence of any commercial or financial relationships that could be construed as a potential conflict of interest.

## Abbreviations

ALL: acute lymphoblastic leukemia
ARID: AT-rich interactive domain
ARIEL: ARID5B inducing enhancer associated long non-coding RNA
B-ALL: B-cell acute lymphoblastic leukemia
BPH: benign prostate hyperplasia
CLL: chronic lymphocytic leukemia
EAL: early age leukemia
ESC: embryonic stem cell
GC: gastric cancer
GECN: geneticand-epigenetic cell cycle network
GWAS: genome wide association study
H3K9Me2: dimethylated Lys 9 on histone H3
HCMV: human cytomegalovirus
HNF4α: hepatocyte nuclear factor 4α
IEG: immediate early genes
LD: linkage disequilibrium
MAPK: mitogen-activated protein kinase
*MLL*: *Mixed Lineage Leukemia*
*MLL-r*: *MLL* rearrangement
MSI: microsatellite instability
MSS: microsatellite stable
MRD: minimal residual disease
MRF2: modulator recognition factor 2
MTX: methotrexate
MTXPG: methotrexate polyglutamate
OR: odds ratio
PCa: prostate cancer
PDGF: platelet-derived growth factor
PHF2: plant homeodomain finger protein
RAF: risk allele frequency
SCNA: somatic copy number alteration
SMC: smooth muscle cell
SNP: single nucleotide polymorphism
T-ALL: T-cell acute lymphoblastic leukemia
TCGA: The cancer Genome Atlas
TEAD4: TEA domain 4.
